# Development of polycationic amphiphilic cyclodextrin nanoparticles for anticancer drug delivery

**DOI:** 10.3762/bjnano.8.145

**Published:** 2017-07-13

**Authors:** Gamze Varan, Juan M Benito, Carmen Ortiz Mellet, Erem Bilensoy

**Affiliations:** 1 Department of Nanotechnology and Nanomedicine, Graduate School of Science and Engineering, Hacettepe University, Ankara, 06800, Turkey; 2 Institute for Chemical Research, CSIC - University of Sevilla, Av. Américo Vespucio 49, Sevilla, 41092, Spain; 3 Department of Organic Chemistry, University of Sevilla, C/ Prof García Gonzalez 1, Sevilla, 41012, Spain; 4 Department of Pharmaceutical Technology, Faculty of Pharmacy, Hacettepe University, Ankara, 06100, Turkey

**Keywords:** amphiphilic cyclodextrin, anticancer, nanoparticle, paclitaxel, polycationic

## Abstract

**Background:** Paclitaxel is a potent anticancer drug that is effective against a wide spectrum of cancers. To overcome its bioavailability problems arising from very poor aqueous solubility and tendency to recrystallize upon dilution, paclitaxel is commercially formulated with co-solvents such as Cremophor EL® that are known to cause serious side effects during chemotherapy. Amphiphilic cyclodextrins are favored oligosaccharides as drug delivery systems for anticancer drugs, having the ability to spontaneously form nanoparticles without surfactant or co-solvents. In the past few years, polycationic, amphiphilic cyclodextrins were introduced as effective agents for gene delivery in the form of nanoplexes. In this study, the potential of polycationic, amphiphilic cyclodextrin nanoparticles were evaluated in comparison to non-ionic amphiphilic cyclodextrins and core–shell type cyclodextrin nanoparticles for paclitaxel delivery to breast tumors. Pre-formulation studies were used as a basis for selecting the suitable organic solvent and surfactant concentration for the novel polycationic cyclodextrin nanoparticles. The nanoparticles were then extensively characterized with particle size distribution, polydispersity index, zeta potential, drug loading capacity, in vitro release profiles and cytotoxicity studies.

**Results:** Paclitaxel-loaded cyclodextrin nanoparticles were obtained in the diameter range of 80−125 nm (depending on the nature of the cyclodextrin derivative) where the smallest diameter nanoparticles were obtained with polycationic (PC) βCDC6. A strong positive charge also helped to increase the loading capacity of the nanoparticles with paclitaxel up to 60%. Interestingly, cyclodextrin nanoparticles were able to stabilize paclitaxel in aqueous solution for 30 days. All blank cyclodextrin nanoparticles were demonstrated to be non-cytotoxic against L929 mouse fibroblast cell line. In addition, paclitaxel-loaded nanoparticles have a significant anticancer effect against MCF-7 human breast cancer cell line as compared with a paclitaxel solution in DMSO.

**Conclusion:** According to the results of this study, both amphiphilic cyclodextrin derivatives provide suitable nanometer-sized drug delivery systems for safe and efficient intravenous paclitaxel delivery for chemotherapy. In the light of these studies, it can be said that amphiphilic cyclodextrin nanoparticles of different surface charge can be considered as a promising alternative for self-assembled nanometer-sized drug carrier systems for safe and efficient chemotherapy.

## Introduction

Paclitaxel (PCX) is an effective wide-spectrum anticancer agent which is isolated from the bark of the tree *Taxus brevifolia* and further obtained semi-synthetically [1]. Its unique antimitotic mechanism depends on inducing the microtubule stabilization and inhibiting the depolymerization of microtubules [2]. PCX binds to N-terminal 31 amino acids of the β-tubulin proteins in microtubules and stabilizes (instead of inhibiting) microtubule assembly to prevent cell division. On the other hand, PCX causes cells to remain in G2/M phase. Microtubules formed by the action of PCX are also dysfunctional and cause cell death [3]. In spite of its promising antitumor activity, the drug has presented considerable difficulties related to its intravenous administration to patients. The most important of these challenges is the very low solubility of PCX in water (0.3 µg/mL) [4]. To overcome poor solubility of PCX in water, the current commercial injectable formulation consists of a 1:1 mixture of anhydrous ethanol and Cremophor EL^®^, which is known to be the cause of severe side effects including nephrotoxicity, neurotoxicity and hypersensitivity reactions [5–6]. Other major problems encountered in the clinical administration of PCX are rapid recrystallization of the drug as a result of dilution in isotonic saline or dextrose solution, leading to severe necrosis and pain at injection site as well as reported incompatibility with intravenous (iv) infusion sets [7]. In order to overcome these side effects of PCX in clinical applications, alternative approaches are developed and evaluated to increase safety and efficacy of chemotherapy with PCX.

A promising step was taken with the FDA approval of albumin nanoparticle bound PCX (Abraxane^®^) in 2005 for breast cancer treatment with a significantly lower dose [8]. This was considered a breakthrough in PCX formulation development as it avoided the use of solubilizers, delivering the drug bound to the nanocarriers in a considerably lower dose to target tissue.

Cyclodextrins (CDs) are cyclic oligosaccharides obtained through enzymatic degradation of starch. The most frequently used CDs in the pharmaceutical field are α-CD, β-CD and γ-CD having 6, 7 and 8 subunits, respectively [9]. These molecules have drawn attention as drug carrier systems for several years because of their unique molecular structures and supramolecular capabilities. CDs, although hydrophilic in the external surface, have hydrophobic cavity and this compartment allows them to form strong inclusion complexes with non-polar drugs or active molecules [10]. CDs are easily able to modulate physicochemical properties of guest molecules, including solubility and/or stability in biological medium. Despite all the advantages, CDs have some challenges. For instance, it is well known that β-CD has low solubility in water and causes haemolysis on blood cells when administered parenterally [11–12]. To overcome these challenges, natural CDs are modified with different chemical groups to alter their structure and improve their biocompatibility [13–16].

Amphiphilic CDs have been synthesized to overcome problems of natural CDs which enhance the interaction with drug molecules and biological membranes [17–18]. Most importantly, amphiphilic CDs possess the ability to spontaneously form nanoparticles at the interface, depending on the preparation method and physical and chemical properties of CD [19–22]. In the literature, amphiphilic CDs were reported to spontaneously self-assemble in the form of nanospheres or nanocapsules and overcome haemolytic activity on blood cells for eventual injectable nanoparticulate drug delivery [23–25].

**Figure 1:**
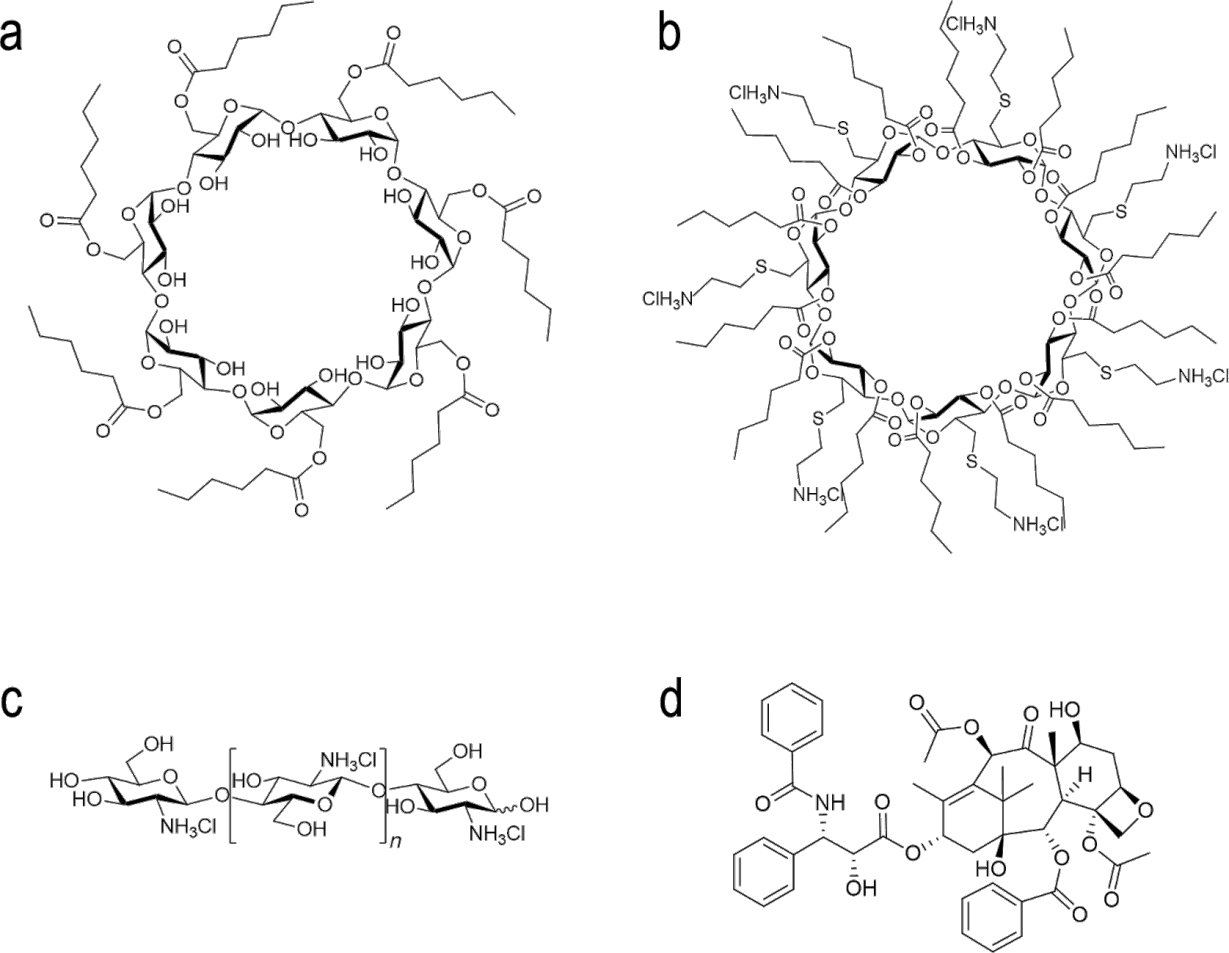
Schematic representation of amphiphilic 6OCaproβCD (a), amphiphilic PC βCDC6 (b), chitosan (c) and paclitaxel (d).

The aim of this study was to evaluate and compare the potential of polycationic amphiphilic CD nanoparticles as delivery systems for effective and safe delivery of PCX in comparison to its non-ionic or core–shell analogues. For this reason, two different cyclodextrin derivatives were used in this context, namely the non-ionic 6OCaproβCD (*M*_W_: 1813 g/mol) (Figure 1a) and the polycationic PC βCDC6 (3178 g/mol) (Figure 1b). 6OCaproβCD is non-ionic as no charged groups are present in the structure in the normal pH window (2–13) and it was used to prepare negatively charged nanoparticles. 6OCaproβCD possesses 7 lipophilic groups on the primary face whilst the polycationic PC βCDC6 has 7 cationic groups on the primary face and 14 lipophilic groups on the secondary face. Both nanoparticles were prepared by a nanoprecipitation technique which is based on spherical crystallites of the polymer while precipitation occurs at the interface. In addition, chitosan (Figure 1c) was used to coat the surface of the 6OCaproβCD nanoparticles. Chitosan-coated 6OCaproβCD nanoparticles (CS-6OCaproβCD) were also prepared and characterized. It was aimed to increase the efficacy of PCX (Figure 1d) as a model drug. All blank amphiphilic CD nanoparticles were optimized for selection of organic solvent, ratio of organic phase to aqueous phase and surfactant concentration to obtain monodisperse particles with a diameter range around 80 to 125 nm. Intended as chemotherapeutic nanocarriers, various PCX-loaded amphiphilic CD nanoparticles were also evaluated for their drug encapsulation, release profile and anticancer activity on MCF-7 human breast cancer cell line in particular. Safety and apoptotic efficacy of blank and PCX-loaded cationic or anionic amphiphilic CD nanoparticles were evaluated with cell culture studies against a series of healthy and cancer cells.

The amphiphilic, cationic PC βCDC6 derivative was used as the anticancer drug carrier delivery system for PCX for the first time in this study. There are various studies in which this derivative is used as a gene transfer delivery system; however, there is only example where this derivative was used as a drug delivery system. This was a study regarding the non-polar anxiolytic drug diapezam realized by Mendez-Ardoy et al. [22]. Our goal is to evaluate the potential of the polycationic CD nanoparticles as an anticancer drug delivery system. In fact, these polycationic CDs were evaluated for their intrinsic apoptotic effect in our first paper [26] in unloaded blank nanoparticle form. This study focuses on the nanocarrier properties and drug delivery system potential of the polycationic CD nanoparticles for PCX, which is an anticancer drug with several serious bioavaibility and toxicity problems. PCX was selected as the target drug in this study also for the fact that it is available on the market in nanomedicine form, known as Abraxane^®^.

## Results and Discussion

### Pre-formulation studies

Nanoparticles are promising carriers for drugs due to their tunable dimensions and shape. There are several factors that influence the particle size, particle distribution, surface charge, homogeneity and shape of nanometer-sized drug delivery systems. These factors have a subsequent influence on the biodistribution and the fate of the nanomedicine in the body [27]. In this case, the formulation parameters play an important role on the mean diameter of the nanoparticles. Our primary concern was to obtain an optimal particle size distribution with a diameter less than 200 nm and a polydispersity index lower than 0.2; therefore, the corresponding parameters were thoroughly assessed.

The effect of different organic solvents used in the organic phase on the mean particle size and polydispersity index (PDI) of blank amphiphilic CD nanoparticles is given in Table 1. It is clearly seen that among the various water-miscible solvents (required for the nanoprecipitation technique), ethanol is the optimal solvent in this study in terms of mean diameter and PDI for all CD nanoparticle formulations. The nanoprecipitation method is mainly based on interfacial turbulence between a miscible organic phase and an aqueous phase [28]. In nanoprecipitation, the polymer and drug is dissolved in a water-miscible organic solvent, which diffuses from the organic phase into the aqueous phase. Meanwhile, polymers in the organic phase tend to spontaneously aggregate, forming spherical crystals, and thus nanoparticles form rapidly [27,29].

**Table 1:** Effect of organic solvent on mean particle size, PDI and zeta potential values of formulations (CD amount is 0.5 mg/mL in all formulations) (*n* = 3, ± standard deviation (SD)).

Nanoparticle formulations	Solvent	Particle diameter ± SD (nm)	PDI ± SD	Zeta potential (mV) ± SD

6OCaproβCD	acetone	164 ± 5	0.62 ± 0.05	−26 ± 2.9
	ethanol	104 ± 1	0.13 ± 0.02	−24 ± 0.3
	methanol	367 ± 2	0.15 ± 0.03	−26 ± 1.4

CS-6OCaproβCD	acetone	285 ± 5	0.34 ± 0.06	+57.2 ± 2.3
	ethanol	122 ± 4	0.23 ± 0.03	+69.1 ± 1.6
	methanol	399 ± 2	0.35 ± 0.03	+61 ± 3.1

PC βCDC6	acetone	124 ± 4	0.32 ± 0.05	+76 ± 0.2
	ethanol	75 ± 2	0.16 ± 0.02	+61 ± 1.4
	methanol	121 ± 6	0.51 ± 0.02	+65 ± 1.3

As seen in Table 1, the mean particle size of the nanoparticles varies greatly in the range between 75 to 400 nm for different solvents, and ethanol gives the smallest diameter for all CD nanoparticles. The effect of organic solvent selection on nanoparticle diameter was found to follow the order of methanol > acetone > ethanol for 6OCaproβCD nanoparticles and CS-6OCaproβCD nanoparticles, and acetone > methanol > ethanol for PC βCDC6 nanoparticles. It is worth noting that ethanol also gave the most monodisperse particles with an acceptable polydispersity index (<0.2) (Table 1).

As expected, the core–shell nanoparticles CS-6OCaproβCD had the largest size due to the chitosan coating on its surface, and the PC βCDC6 nanoparticles were the smallest, probably resulting from the likely electrostatic destabilization of larger particles.

As is known, nanoparticle homogeneity is based on the properties of the organic solvent in the nanoprecipitation technique. It is shown that ethanol is the optimum organic solvent for amphiphilic CDs in this study. In the nanoprecipitation technique, nanoparticle formation occurs as a result of interfacial turbulence between two unequilibrated liquid phases. For the formation of turbulence, the liquid phases (organic phase and liquid phase) used in this method must be miscible with each other. Galindo-Rodriguez et al. investigated the influence of the different solvent types on NP formation in the nanoprecipitation technique [30]. The solvent and solubility parameters were calculated by using the dispersion force component, the polar component, and the hydrogen bonding component. It was reported that the smaller the difference between the solubility of solute and solvent, the higher the affinity and the smaller the particle size. They emphasized that the difference in polarity between ethanol/water is the smallest compared to the difference between the other solvents/water, and the smallest particle size is obtained in the formulation using ethanol [30]. In another study, Khan et al. prepared gelatine nanoparticles by the nanoprecipitation technique with different organic solvents (methanol, ethanol, acetone, *n*-propanol and acetonitrile) concluding that only methanol and ethanol led to nanometer-sized particles among those solvents that were studied. Furthermore, ethanol was reported to provide the smallest particle size (250 nm) between these two organic solvents [31] in parallel to the findings presented in Table 1.

As another major parameter influencing particle formation and size, the effect of surfactant presence and concentration was determined by investigating the mean particle size of amphiphilic CD nanoparticles for 0, 0.1 and 0.5% w/v pluronic F68 (PF68) dissolved in aqueous phase. Table 2 shows that the mean particle size increases in proportion with concentration of PF68.

**Table 2:** Effect of surfactant concentration on nanoparticle diameter and dispersity in ethanol (CD amount is 0.5 mg/mL in all formulations) (*n* = 3, ± SD).

Nanoparticle formulations	PF68 concentration (% w/v)	Particle diameter ± SD (nm)	PDI ± SD

6OCaproβCD	0	104 ± 1	0.13 ± 0.02
	0.1	190 ± 4	0.17 ± 0.03
	0.5	208 ± 5	0.23 ± 0.02

CS-6OCaproβCD	0	122 ± 4	0.23 ± 0.03
	0.1	168 ± 6	0.15 ± 0.03
	0.5	185 ± 4	0.33 ± 0.06

PC βCDC6	0	75 ± 2	0.16 ± 0.02
	0.1	110 ± 7	0.37 ± 0.01
	0.5	175 ± 5	0.47 ± 0.04

The smallest particle size was obtained without the surfactant for all nanoparticle formulations. This is found to be in accordance with previous studies reported in the literature proving that amphiphilic CDs are able to form nanoparticles without the presence of surfactants [21–22,24,32–34] due to their favorable self-alignment properties at air–water or oil–water interface [35]. The mean particle size of amphiphilic CD nanoparticles increased linearly with concentration of surfactant. Bilensoy et al. evaluated the effect of the presence of PF68 in CD nanoparticle formulations on cytotoxicity on L929, a healthy mouse fibroblast cell line. According to these results, it was suggested that PF68 has no significant effect on size and drug loading capacity of nanoparticles but dose-dependent toxicity could occur on L929 fibroblast cells [36]. In another study, a polycationic, amphiphilic, cyclodextrin derivative was used to prepare nanospheres and nanocapsules as drug delivery systems. When the results are compared with this study in terms of particle size, it can be concluded that the use of surfactant is linearly correlated with the particle size [22].

### Characterization of PCX-loaded amphiphilic CD nanoparticles

According to pre-formulation studies described and discussed in the previous section, it was decided that the most suitable solvent is ethanol for all CD formulations. Each PCX-loaded nanoparticle formulation was prepared with ethanol and without any surfactant (PF68).

Delivering the therapeutic load to the target site and maintaining therapeutic blood levels for the drug in an effective dose is the most important objective for targeted nanomedicines. Drug encapsulation efficiency is highly affected by the nature of the polymer/polysaccharide used to prepare the nanoparticles. Therefore, in order to determine the effect of surface charge on drug loading capacity of nanoparticles, PCX was chosen as a model anticancer drug frequently used in chemotherapy for patients with breast cancer. The encapsulation efficiency of amphiphilic CD nanoparticles is given in Table 3. The quantity of loaded PCX was determined directly with a validated HPLC method and entrapment efficiency or associated drug percentage were calculated with Equation 1 or Equation 2, as described later in the Experimental section. As seen in Table 3, the drug loading capacity of the nanoparticles was strongly related to the surface charge of the CD nanoparticles. As is known, PCX itself is negatively charged, so encapsulation due to electrostatic interactions is favored for the cationic CD nanoparticles, CS-6OCaproβCD and PC βCDC6, resulting in a 1.5-fold higher loading for this drug in cationic nanoparticles compared to the negatively charged 6OCaproβCD nanoparticles as seen in Table 3.

**Table 3:** Associated drug (%) and entrapment drug quantity (µg/mg) of amphiphilic CD nanoparticles for PCX (CD amount is 0.5 mg/mL and initial PCX amount is 0.05 mg/mL in all formulations) (*n* = 3, ± SD).

Nanoparticle formulations	Percentage associated drug ± SD	Entrapment drug quantity ± SD (µg/mg)

6OCaproβCD	41 ± 2	4.4 ± 0.4
CS-6OCaproβCD	62 ± 5	5.6 ± 1.3
PC βCDC6	64 ± 2	6.3 ± 0.7

According to these results, the CS coating increased drug loading capacity of anionic 6OCaproβCD nanoparticles by approximately 50%. In addition, the CS coating may provide more efficient encapsulation area for PCX from aqueous media. It can be said that this hypothesis is also valid for PC βCDC6 nanoparticles. This amphiphilic CD derivative has long aliphatic chains terminated with amine groups. PC βCDC6 nanoparticles are believed to encapsulate PCX not only in the hydrophobic cavity but also between the long cationic aliphatic chains of the cyclodextrin as PCX and CD are co-nanoprecipitated during the preparation method.

Table 4 shows the final mean particle size, PDI and zeta potential values of PCX-loaded amphiphilic CD nanoparticles. The mean diameter of PCX-loaded nanoparticles varies in the range of 82 to 125 nm according to the type of CD used. They also exhibit a narrow distribution as the preparation technique nanoprecipitation was kept standard for all formulations.

**Table 4:** Mean particle size, PDI and zeta potential of PCX-loaded nanoparticles (CD amount is 0.5 mg/mL and initial PCX amount is 0.05 mg/mL in all formulations) (*n* = 3, ± SD).

Nanoparticle formulations	Particle size ± SD (nm)	PDI ± SD	Zeta potential ± SD (mV)

6OCaproβCD	113 ± 4	0.13 ± 1	−29 ± 2
CS-6OCaproβCD	125 ± 2	0.22 ± 4	+44 ± 3
PC βCDC6	82 ± 2	0.16 ± 5	+62 ± 1

In addition, drug loading did not cause significant changes in mean diameter of the nanoparticles except that an increase in diameter was observed for all nanoparticles. This suggests that the drug is partially adsorbed as a layer on the nanoparticle surface and partially encapsulated in the matrix due to charge interactions since PCX is a molecular entity with a carboxilic acid end, thereby anionic at neutral pH. Although the differences between the particle sizes of the blank and drug-loaded nanoparticles are not statistically significant, the smallest difference is seen in the CS-coated nanoparticles. The difference between the particle sizes of the blank and drug-loaded nanoparticles may be related to the localization of the drug. When the nanoparticles were prepared, the drug and cyclodextrins were dissolved together in the organic phase. Meanwhile, some of the drug is encapsulated by the hydrophobic cavity of the cyclodextrins and some of the drug is adsorbed on the surface of nanoparticles. This drug on the surface of the nanoparticles changes the particle size. For CS-coated nanoparticles, the drug and cyclodextrin were dissolved in the organic phase and then added to the CS-containing water. The presence of chitosan in the aqueous phase may cause a charge interaction between the adsorbed drug on the surface of the nanoparticles and the chitosan, resulting in a more rigid structure. In another previous study, it was reported that the new amphiphilic CD derivative PC βCDC6 is suitable to form stable nanoparticles with small particle size [26]. The particle size of nanoparticulate drug delivery systems play a direct and important role on cellular uptake, systemic circulation, toxicity and stability of nanoparticles [37–38]. It was reported that nanoparticles smaller than 200 nm can escape recognition by the mononuclear phagocytic system (MPS) [39]. The prolonged circulation time for nanoparticles, *t*, is needed to escape from MPS uptake in order to reach the tumor tissue. The MPS is one of the most important factors in preventing the prolonged circulation, affecting the biodistribution of nanoparticles. In this way, more effective and safe therapy can be provided with lower drug dose.

Zeta potential measurements indicate that 6OCaproβCD has a negative surface charge unlike the other formulations. In this study, PC βCDC6 has a strong positive surface charge owing to polycationic amino groups. This amphiphilic CD derivative was previously used for gene delivery studies due to net positive surface charge, facilitating the condensation of negatively charged DNA to form polyplexes [40–41]. In addition, CS-6OCaproβCD nanoparticles are also positively charged due to coating with cationic polymer. It is known that chitosan is a natural bioactive cationic polysaccharide derived from deacetylation of chitin and is well-characterized for its mucosal penetration enhancer property and apoptotic activity against cancer cells [42]. To alter the surface charge of nanomaterials, chitosan can be used as coating material in nanoparticles [43–44]. As a result of the surface coating with chitosan, the zeta potential value of 6OCaproβCD nanoparticles increased from −29 mV to +44 mV as seen in Table 3. Unal et al., prepared uncoated and CS-coated 6OCaproβCD nanocapsules for oral camptothecin delivery. They reported that the CS coating increased the zeta potential of nanocapsules from −11 to +10 mV [45–46].

Both CS-coated CD and PC βCDC6 were able to render a net positive charge to the nanoparticles while 6OCaproβCD had a charge around −25 mV. Nanoparticles with zeta potential between −10 and +10 mV are classified as neutral. Nanoparticles with zeta potential greater than +30 mV and less than −30 mV are considered as strongly charged [47]. According to this classification, two net positive nanoparticle formulations and a net negative nanoparticle formulation were used as a nanometer-sized drug delivery system for PCX in this study. These differences between the surface charge of CD nanoparticles allowed the comparison of the effect of surface charge on drug loading capacity, stability and anticancer activity in this study.

**Figure 2:**
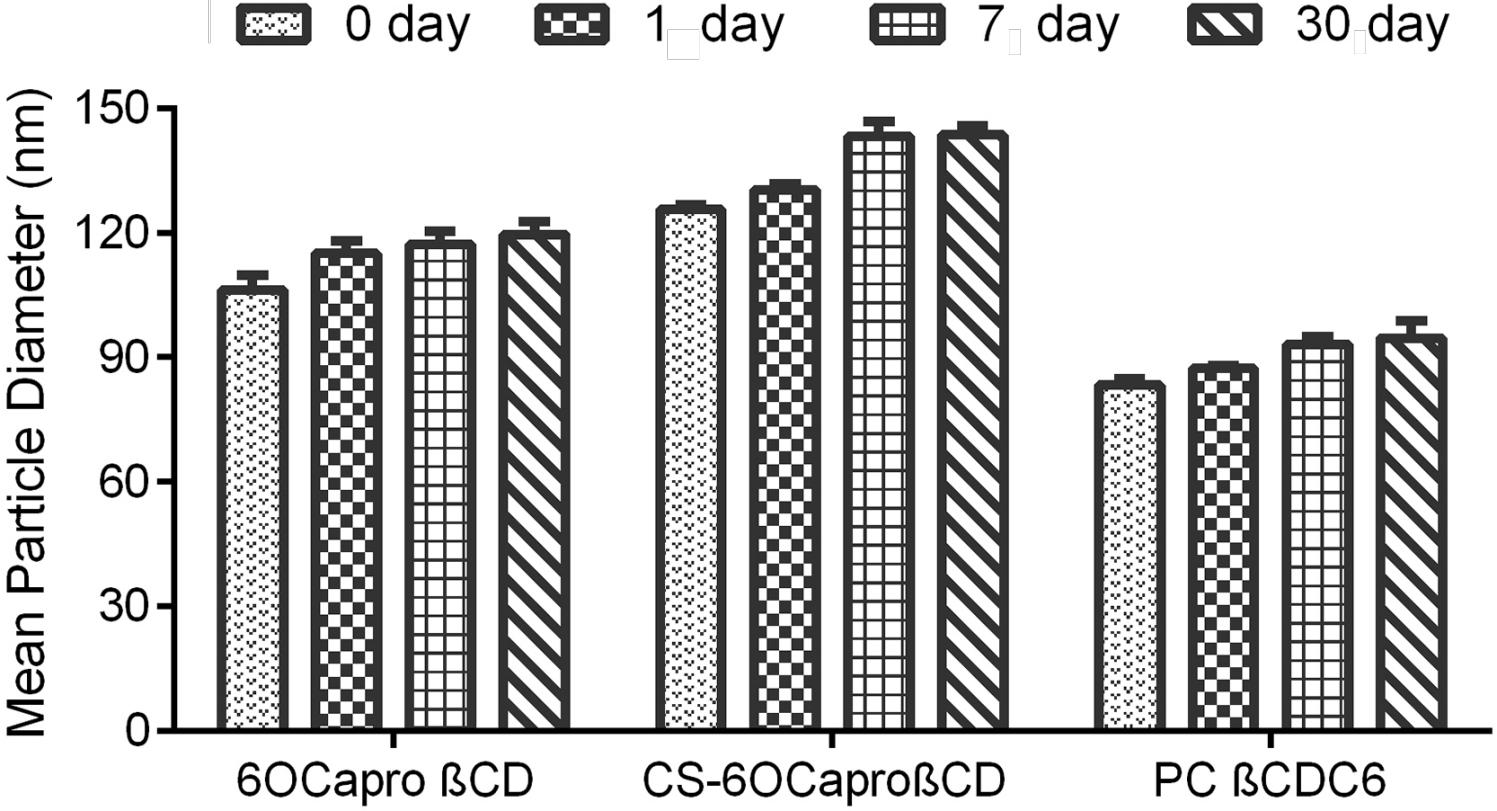
Time-dependent variation of particle size (nm) of PCX-loaded amphiphilic CD nanoparticles stored in aqueous dispersion form, (*n* = 3, ± SD).

**Figure 3:**
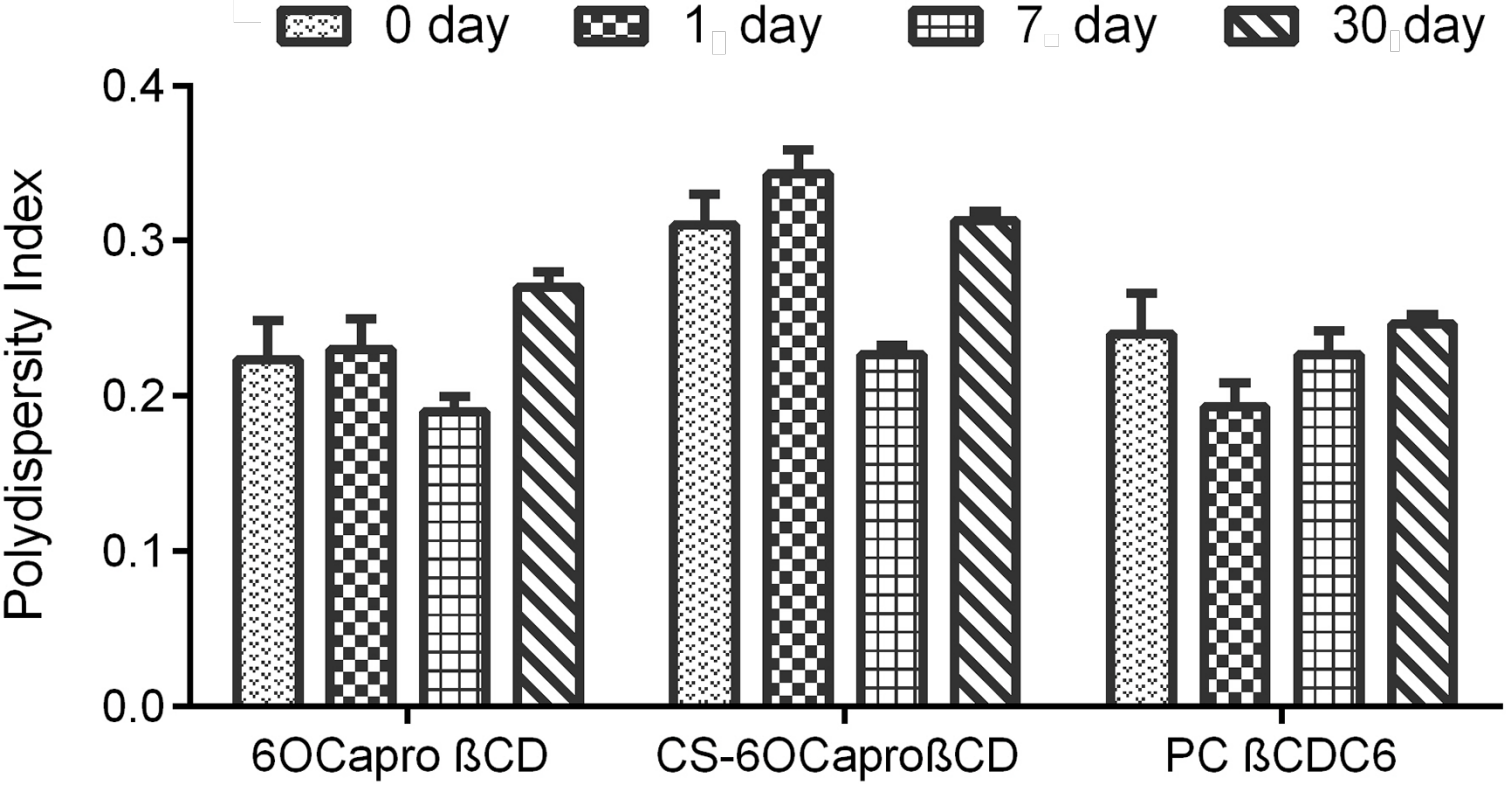
Time-dependent variation of the PDI value of PCX-loaded amphiphilic CD nanoparticles stored in aqueous dispersion form (*n* = 3, ± SD).

**Figure 4:**
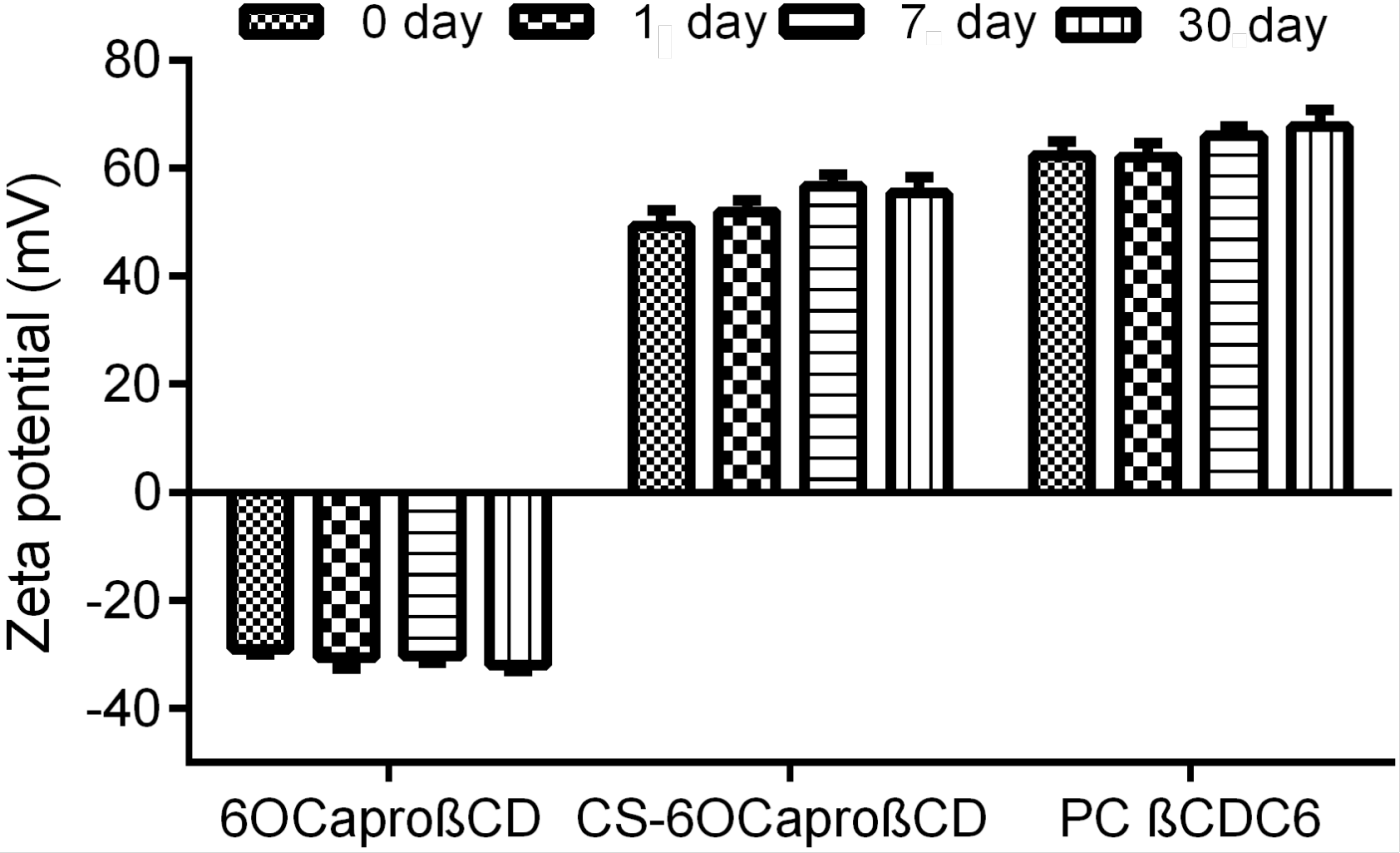
Time-dependent variation of the zeta potential value of PCX-loaded amphiphilic CD nanoparticles stored in aqueous dispersion form (*n* = 3, ± SD).

Furthermore, mean particle size distributions and PDI of the blank and PCX-loaded nanoparticles were followed for one month in aqueous form to determine the physical stability of PCX-loaded amphiphilic CD nanoparticle dispersions. Figure 2, Figure 3 and Figure 4 show that there is no significant difference for particle size, PDI and zeta potential of PCX-loaded and blank CD nanoparticle formulations (*p* > 0.05). PCX-loaded nanoparticles maintained their stability for 30 days in ultrapure water. This data shows that PCX crystals are not formed in aqueous dilution, which is believed to improve the safety of the drug delivery system.

PCX exists in a crystal form in aqueous media due to hydrophobic interaction between lipophilic groups [48–49]. Due to this phenomenon, PCX is recrystallized in minutes as a result of dilution in isotonic saline solution for intravenous (iv) infusion, which is the preferred delivery route for chemotherapy. This is one of the main problems of clinical application of PCX. In the light of the physical stability studies depicted in Figures 2–4, it can be said that all amphiphilic CD nanoparticles maintained PCX in dispersed form within their hydrophobic matrix and thus, ensured stability of drug in aqueous media, which is also supported by previous studies for 6OCaproβCD nanocapsules and nanospheres [24].

**Figure 5:**
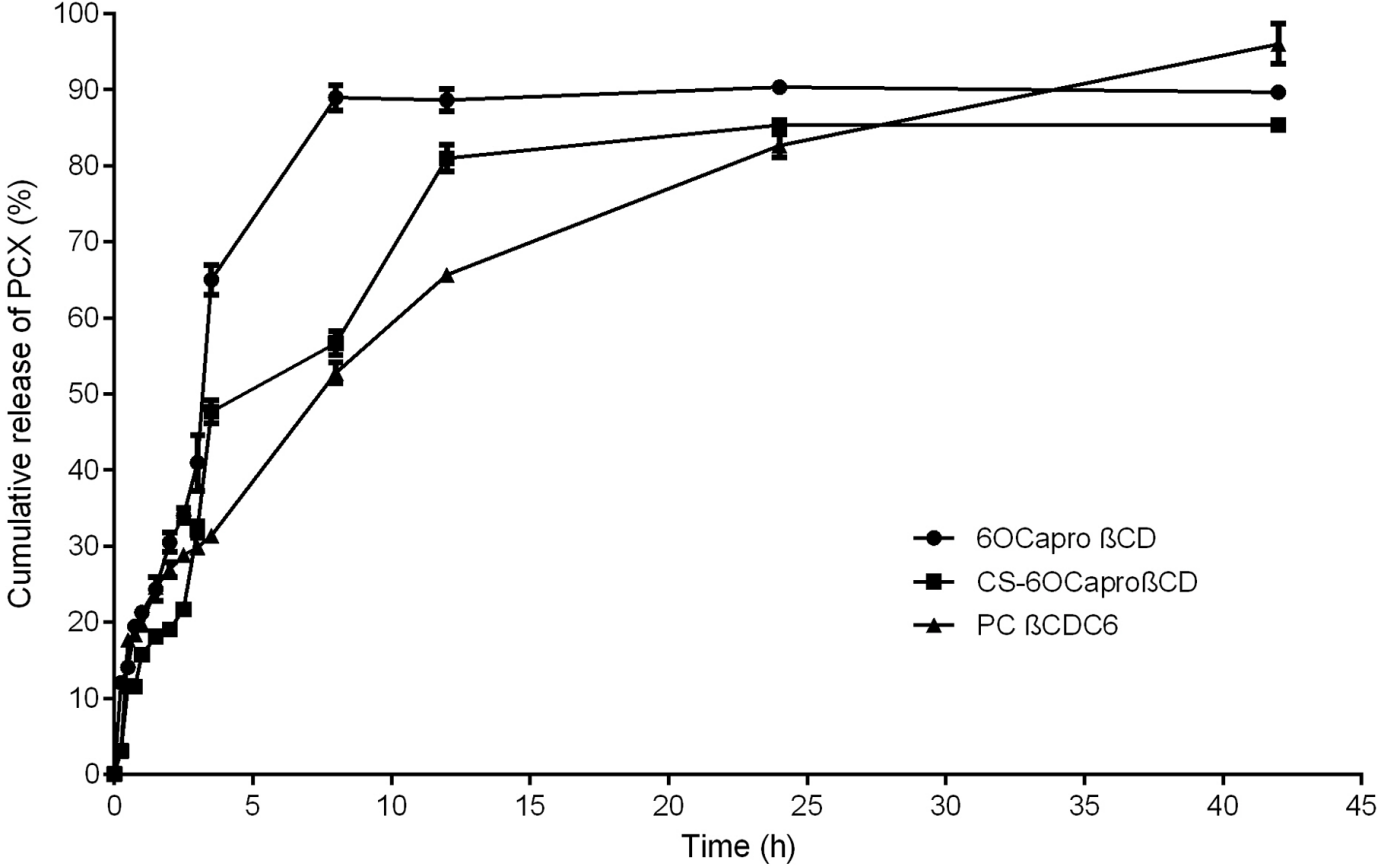
Cumulative release profile of PCX from different amphiphilic CD nanoparticles at pH 7.4 phosphate buffer solution under sink conditions (*n* = 3, ± SD).

The in vitro release profile of PCX from CD nanoparticles was determined using the dialysis bag method with HPLC as detailed in the Experimental section. As seen in Figure 5, PCX release from PC βCDC6 exhibited a markedly slower release profile of up to 42 h compared with other formulations. The release profiles indicated that in the first 5 h approximately 50% of PCX was released from the CS-6OCaproβCD and 70% from anionic 6OCaproβCD nanoparticles formulations, which can be attributed to desorption of surface PCX. Meanwhile, a 50% release time for PCX was found to be 8 h from PC βCDC6 nanoparticles. In addition, the release profile of PCX was found to reach plateau levels at 8, 12 and 42 h for 6OCaproβCD, CS-6OCaproβCD and PC βCDC6 nanoparticles, respectively.

PCX carries a negative charge and therefore has a stronger interaction with the positively charged CD, thus PCX release from PC βCDC6 is slower than other formulations. The CS coating of 6OCaproβCD nanoparticles also relatively slows down the release. However, the core–shell approach is believed to be insufficient to prolong the release of PCX as a result of both the hydrophobic nanoparticle matrix and the strong positive charge due to the negative charge of PCX.

It was reported in the literature that large nanoparticles result in a slower release profile than smaller nanoparticles [50]. However, in this study, PC βCDC6 nanoparticles have the smallest particle size and the longer release profile, as seen in Figure 5. It can therefore be suggested that the surface charge of nanoparticle is directly effective on the drug release profile.

### Cell culture studies

In order to determine the safety of blank amphiphilic CD nanoparticles and the anticancer efficacy of PCX-loaded amphiphilic CD nanoparticles, L929 mouse fibroblast cells and MCF-7 human breast cancer cell lines were used, respectively. Both cell lines were grown and incubated in appropriate conditions (see Experimental section for full experimental details).

**Figure 6:**
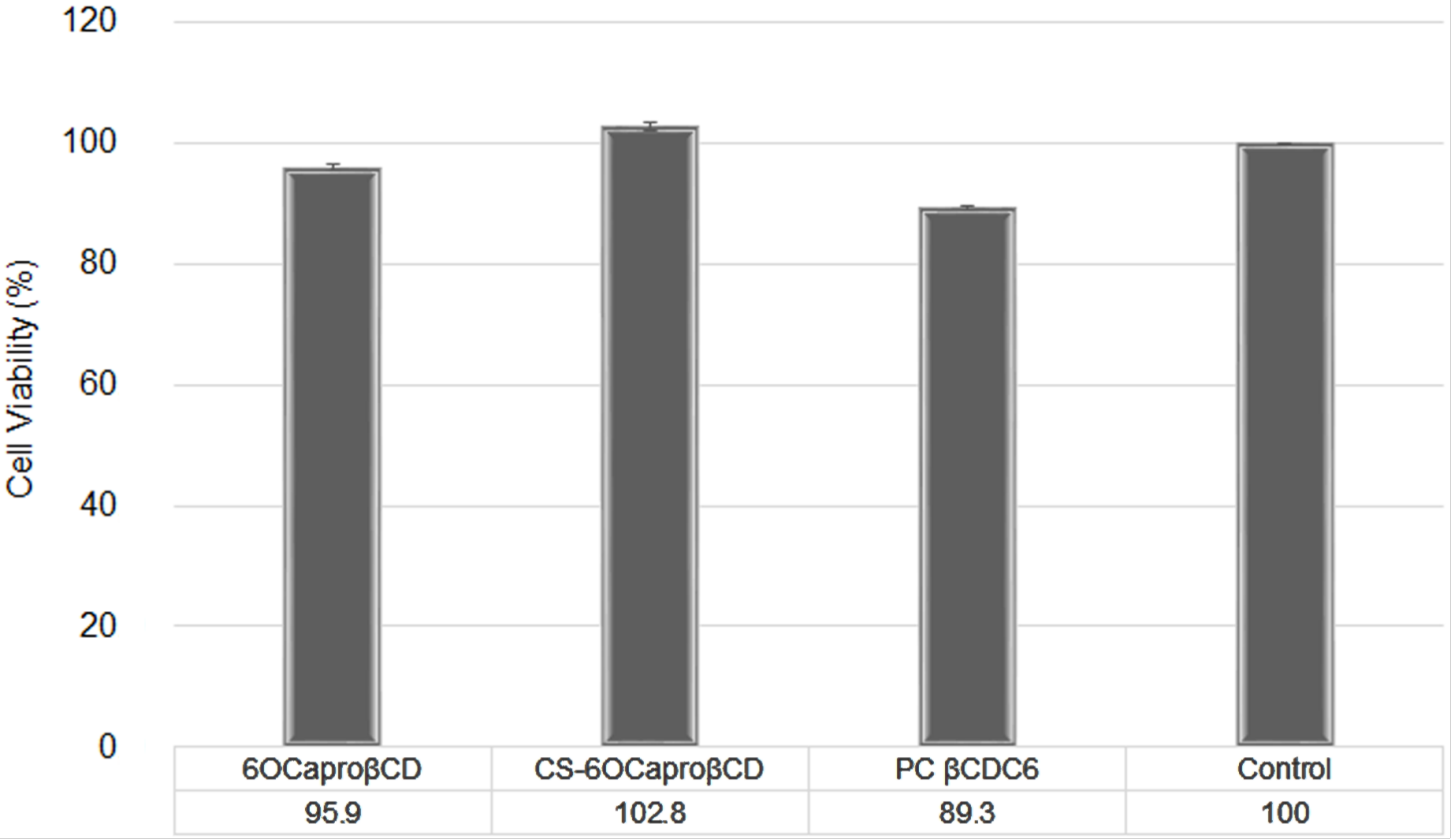
Cytotoxicity of unloaded amphiphilic CD nanoparticles on L929 mouse fibroblast cell line with MTT assay (CD concentration is 0.5 mg/mL in all formulation) (*n* = 3, ± SD).

The cytotoxicity of blank amphiphilic CD nanoparticles was determined on L929 mouse fibroblast cells with MTT assay. This cell line is recommended by the U.S. Pharmacopeial Convention (USP) for the cytotoxicity evaluation of polymeric systems and was therefore used. According to MTT assay, cell viability for L929 cells is given in Figure 6.

It is clearly shown that all blank amphiphilic CD nanoparticle formulations are non-cytotoxic on L929 fibroblast cells compared with the control group (*p* > 0.05). It can therefore be concluded that blank amphiphilic CD nanoparticles have no cytotoxic effect on healthy cells. It was previously reported that toxicity of blank amphiphilic CD nanocapsules and nanospheres are concentration dependent and that they are also non-hemolytic [24,45]. Therefore, these nanoparticles may be safe on healthy cells as drug carrying systems.

**Figure 7:**
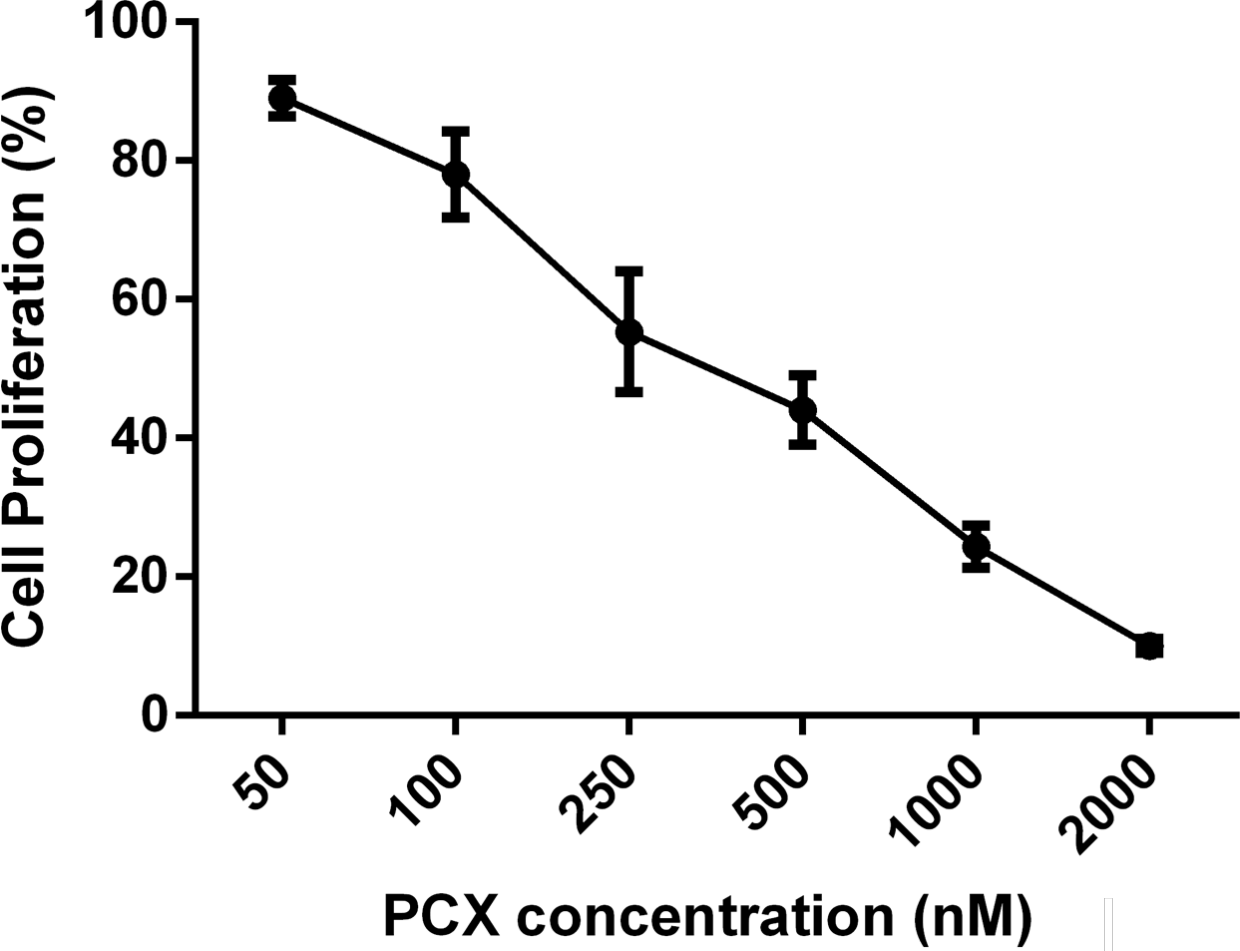
IC_50_ value of PCX solution in DMSO on MCF-7 human breast cancer cell line (*n* = 3, ± SD).

To optimize the concentration of CD nanoparticles for cell culture studies, the inhibitory concentration 50 (IC_50_) value of PCX was calculated on MCF-7 human breast cancer cell line. For this purpose, MCF-7 cells were incubated with different concentrations of PCX in dimethyl sulfoxide (DMSO). Non-treated cells were incubated with DMEM alone and were used as control group. Cell proliferation was determined and the IC_50_ value of PCX was calculated and the results are given in Figure 7.

As seen in Figure 7, the IC_50_ of PCX is 250 nM for the MCF-7 cell line. This result agrees with the literature [51]. According to the IC_50_ study results, nanoparticles loaded with 250 nM PCX were further used for cell culture studies.

**Figure 8:**
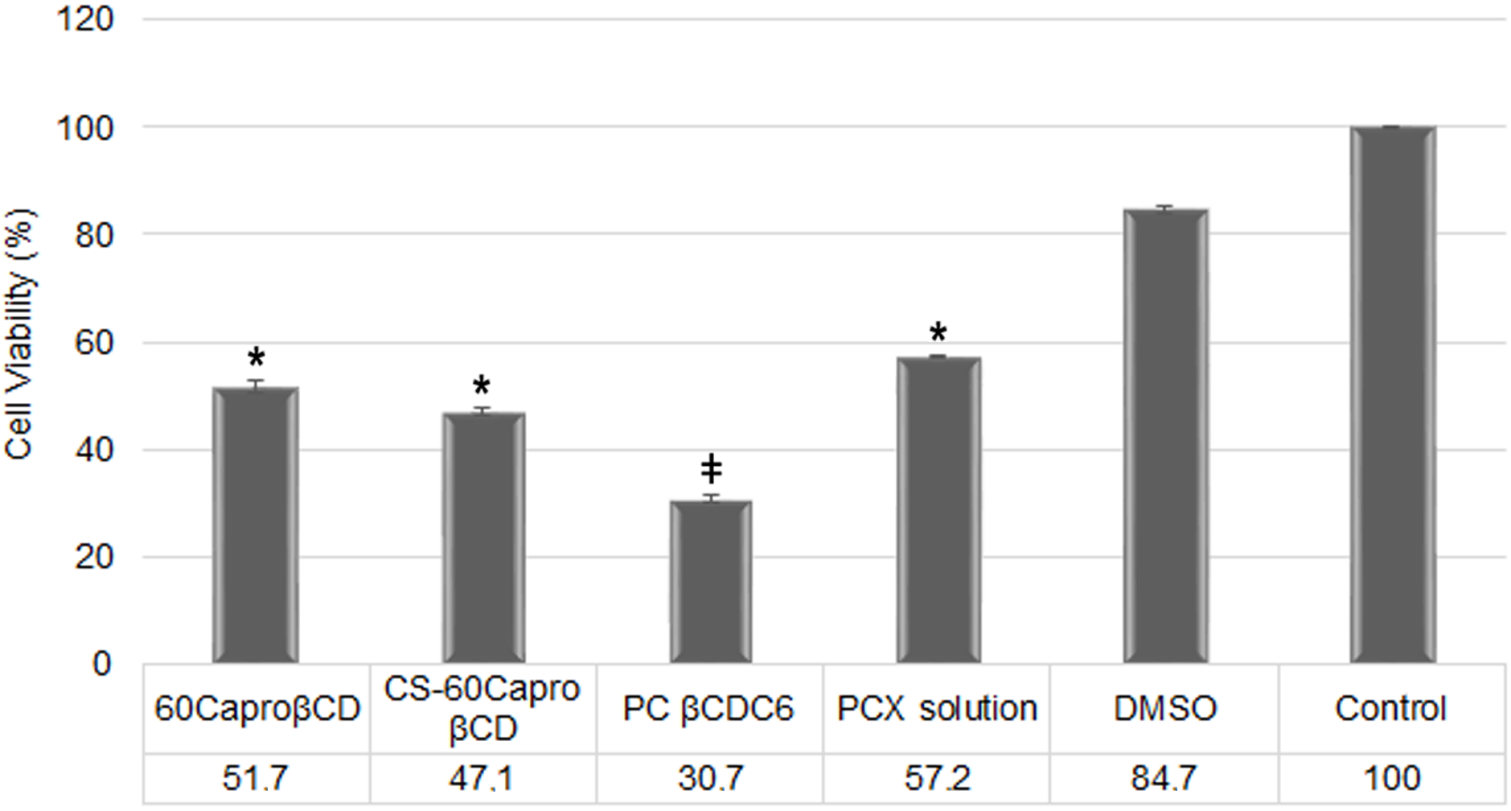
Anticancer activity of PCX-loaded amphiphilic CD nanoparticle formulations and PCX solution in DMSO on MCF-7 human breast cancer cell line after 48 h of incubation (All CD nanoparticle formulations and PCX solution contain 250 nM PCX) (*n* = 3, ± SD). Note that: * *p* < 0.05 as compared with the control, and ╪ *p* < 0.05 as compared with other CD nanoparticle formulations.

The anticancer activity of PCX-loaded nanoparticles was determined on MCF-7 cell lines. After an incubation period, cell viability was calculated, as shown in Figure 8.

According to the results of anticancer activity studies on MCF-7, PCX-loaded amphiphilic CD nanoparticles have higher cytotoxicity than PCX solution in DMSO (*p* < 0.05). The amphiphilic CD nanoparticles and the drug solution carry an equivalent amount of PCX (250 nM) during the cell culture study. The cell viability in loaded CD nanoparticles is significantly different from the PCX solution (*p* < 0.05). Moreover, the effect of surface charge on viability of cancer cells can be clearly seen in Figure 8. Anticancer activity increases with increasing surface charge of nanoparticles. It was known that the cell membrane is negatively charged so that cationic nanoparticles enhance interaction with the biological membrane. Positively charged nanoparticles can bind with negatively charged molecules (e.g., sialic acid, cholesterol, phospholipid) on cell membrane easier than anionic nanoparticles [26,52]. In addition, the surface charge of nanoparticles play an important role on cellular uptake and subcellular localization [53–54]. Another reason for the cell viability differences of CD nanoparticles may be related with drug release profiles. PCX shows anticancer activity by stabilizing microtubules and blocking the cell in G2 or M phase in cell cycle [55–56]. The duration of drug release of PCX-loaded amphiphilic CD nanoparticles increases in the order of 6OCaproβCD < CS-6OCaproβCD < PC βCDC6. Therefore, the amphiphilic CD nanoparticles carried different drug amounts when they were taken up by MCF-7 cells. This can explain the difference in the cell viability between CD nanoparticle formulations.

## Conclusion

In this study, 6OCaproβCD, CS-6OCaproβCD and PC βCDC6 nanoparticles were prepared and used as nanometer-sized delivery systems and compared in terms of mean particle size, zeta potential, drug loading capacity and drug release profile for PCX, which is an effective anticancer agent over the wide spectrum various types of cancer. The findings strongly suggest that positive charge can improve drug loading capacity, slow down drug release and improve cellular interaction due to the negative charge of the cell membrane. Furthermore, unloaded or loaded nanoparticle cytotoxic effects were demonstrated with MTT assay in this study. In the light of the results of this study, it is clearly demonstrated that anionic and cationic CD nanoparticles are suitable carriers for PCX. Moreover, PC βCDC6 was used to prepare nanoparticulate, anticancer drug delivery systems for the first time in literature. Cationic CD nanoparticles can be considered as promising carriers for PCX as well as other lipophilic anticancer drugs for cancer therapy. In addition, by formulating with anionic and cationic amphiphilic CDs, it will be possible to enhance anticancer activity of drugs, overcoming the problem of surfactant-induced toxicity. Finally, it can be said that polycationic amphiphilic CDs are favorable, nanoparticulate, drug delivery systems for the delivery of anticancer agents.

## Experimental

### Materials

Anionic 6OCapro βCD and PC βCDC6 were synthetized as described previously in University of Sevilla, Spain [26]. PCX (≥97% powder, *M*_W_: 853.91 g/mol) was purchased from Sigma-Aldrich, Germany. The chitosan used for coating the nanoparticles (Protasan UP G-113; *M*_W_: <200 kDa, viscosity: <20 mPa·s), was purchased from Novamatrix, Norway. Cellulose membrane dialysis tubing (avgerage flat width 25 mm, MWCO: 14,000 Da) was purchased from Sigma-Aldrich, Germany. All other chemicals used were of analytical grade and obtained from Sigma-Aldrich. Ultrapure water was obtained from a Millipore Simplicity 185 Ultrapure water system (Millipore, France).

### Methods

#### Preparation of unloaded or PCX-loaded amphiphilic CD nanoparticles

PC βCDC6 nanoparticles and anionic 6OCaproβCD nanoparticles were prepared according to the nanoprecipitation method as described previously [26,28]. Briefly, 1 mg of PC βCDC6 or 6OCaproβCD was dissolved in 1 mL of organic solvent (ethanol, methanol or acetone) (0.1% w/v). This organic phase was added dropwise into aqueous phase (2 mL) containing PF68 (0–0.5% w/v) under magnetic stirring at room temperature. Then, the organic phase was evaporated under vacuum at 40 °C to the desired final volume of 2 mL. To prepare CS-coated 6OCaproβCD nanoparticles, the same technique was employed in the presence of protosan (0.025%, w/v) in the aqueous phase. According to the results of the pre-formulation studies, optimal formulation parameters were selected for PCX-loaded amphiphilic CD nanoparticles. To prepare drug-loaded nanoparticles, PCX (0.1 mg) and cyclodextrin (1 mg) were co-nanoprecipitated in 1 mL organic solvent and then organic phase was poured in 2 mL ultrapure water using the conditions previously given.

#### Mean particle size distribution and surface charge

The mean particle diameter (nm), PDI and zeta potential (mV) of amphiphilic CD nanoparticles were determined by dynamic light scattering (DLS) (NanoZS, Malvern Instruments, UK). All formulations were measured at an angle of 173° for particle size measurements and 12° for zeta potential measurements. All formulations were measured at room temperature in triplicate for thesize and zeta potential analysis.

#### Drug loading capacity and in vitro release profile of PCX-loaded amphiphilic CD nanoparticles

The content of PCX in amphiphilic CD nanoparticle formulations was quantified directly with a validated HPLC method [32] (HP Agilent 1100 HPLC system, Germany). Briefly, PCX-loaded nanoparticle formulations were lyophilized for 24 h following centrifugation at 10,000 rpm for 15 min to remove free PCX. The supernatant was collected and freeze-dried. The lyophilized nanoparticle powder was dissolved in dichloromethane (DCM) to quantify nanoparticle-bound PCX (µg/mL).

The HPLC system consisted of reverse phase C18 column (Hichrom 5, 250 × 4.6 mm, U.K.) and acetonitrile: ultrapure water (70:30 v/v) as a mobile phase was delivered at a flow rate of 1.00 mL/min. A 50 µL aliquot of sample was injected for analysis. PCX was quantified by UV detection (λ = 227.4 nm) at 25 °C. Drug loading was expressed as described in Equation 1 and Equation 2 to clearly express the drug percentage bound to nanoparticles as well as drug entrapped per unit polymer.

[1]
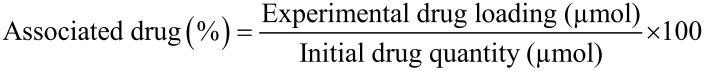


[2]
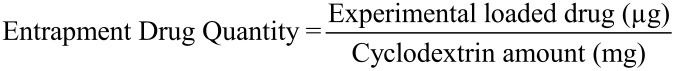


The in vitro cumulative release profile of PCX from CD nanoparticles was determined with the dialysis membrane technique under sink conditions in a shaking water bath at 37 °C in PBS pH 7.4. Briefly, drug-loaded nanoparticle dispersions were added in the dialysis membrane (Sigma, cellulose membrane, MWCO: 100,000 Da, Sigma Chemicals). The nanoparticle-containing dialysis bags, closed with stoppers on both ends, were placed in PBS pH 7.4 containing 0.1% Tween 80 at 37 °C to provide sink conditions. The samples were taken from the medium at specific time intervals and replaced with fresh PBS at the same volume and temperature. The PCX amount in the samples was determined with HPLC as described previously.

#### Physical stability of blank or drug-loaded nanoparticles

In order to determine the physical stability of PCX in the nanoparticles, drug-loaded nanoparticles were stored in ultrapure water at 4 °C and the mean particle size, PDI values and zeta potential were obtained periodically for 30 days in aqueous dispersion form to elucidate whether PCX crystals are formed or any aggregation/precipitation is observed upon storage of the nanoparticle dispersions.

#### Cell culture studies

In order to determine safety or anticancer efficacy of blank amphiphilic CD nanoparticles, L929 mouse fibroblast cells or MCF-7 human breast carcinoma cell lines were used, respectively. Both cell lines were cultured in the same conditions as a monolayer in Dulbecco’s modified Eagle’s medium (DMEM) supplemented with 10% fetal bovine serum (FBS), penicillin (100 units/mL) and streptomycin (100 µg/mL). The cultures were maintained at 37 °C in a humidified 5% CO_2_ incubator. The cell lines were seeded in 96-well tissue culture plates at a density of 1 × 10^3^ cells/well in DMEM (100 µL), separately.

After the L929 cells reached confluence, DMEM was removed from the cells and fresh medium containing blank amphiphilic CD nanoparticles was replaced and incubated for 48 h. In order to determine cell viability, 3-(4,5-dimethylthiazol-2-yl)-2,5-diphenyltetrazolium bromide (MTT) assay was applied. For this purpose, 20 µL of MTT solution in PBS (5 mg/mL) was added in each well and incubated for 4 h. After incubation, 100 mL of DMSO was added per well to dissolve formazan crystals. The optical density (OD) was determined by a microplate reader (Molecular Devices, USA) at 450 nm.

In order to determine the anticancer activity of loaded nanoparticles, the IC_50_ value of PCX was calculated firstly. For this purpose, after the MCF-7 cells reached full confluence, DMEM was replaced with different concentrations of a PCX solution in DMSO (50, 100, 250, 500, 1000 and 2000 nM) and incubated for 48 h. After the incubation time, the MTT assay was applied described above. According to the IC_50_ study, amphiphilic CD nanoparticles were prepared and diluted with DMEM to contain 250 nM PCX. The control group consisted of cells incubated in DMEM alone for two groups and PCX solution in DMSO for the MCF-7 cell line. After that, using MTT assay, the cell viability was determined.

#### Statistical Analysis

All statistical analyses were performed by Student’s *t*-test using GraphPad Prism version 6 (San Diego, CA, USA). A value of *p* < 0.05 was considered to denote a statistically significant difference.
